# Affective Benefits of Nature Contact: The Role of Rumination

**DOI:** 10.3389/fpsyg.2021.643866

**Published:** 2021-03-10

**Authors:** Gregory N. Bratman, Gerald Young, Ashish Mehta, Ihno Lee Babineaux, Gretchen C. Daily, James J. Gross

**Affiliations:** ^1^School of Environmental and Forest Sciences, University of Washington, Seattle, WA, United States; ^2^Department of Psychology, University of California, Berkeley, Berkeley, CA, United States; ^3^Department of Psychology, Stanford University, Stanford, CA, United States; ^4^Senior Behavioral Scientist, Uplight, Boulder, CO, United States; ^5^Department of Biology, Stanford University, Stanford, CA, United States; ^6^Center for Conservation Biology, Stanford University, Stanford, CA, United States; ^7^The Natural Capital Project, Stanford, CA, United States; ^8^Stanford Woods Institute, Stanford University, Stanford, CA, United States

**Keywords:** nature contact, emotion regulation, affect, structural equation analysis, rumination

## Abstract

Mounting evidence shows that nature contact is associated with affective benefits. However, the psychological mechanisms responsible for these effects are not well understood. In this study, we examined whether more time spent in nature was associated with higher levels of positive affect in general, and lower levels of negative affect and rumination in general. We also conducted a cross-sectional mediation analysis to examine whether rumination mediated the association of nature contact with affect. Participants (*N* = 617) reported their average time spent in nature each week, as well as their general levels of positive and negative affect, and the degree to which they typically engaged in rumination in daily life. We then used structural equation modeling to test our hypotheses. Our results support the hypothesis that nature contact is associated with general levels of affect, and that rumination mediates this association for negative affect, and marginally mediates this association for positive affect.

## Introduction

Studies from multiple disciplines support an association of nature contact with psychological well-being (Bowler et al., 2010; Keniger et al., 2013; Frumkin et al., 2017; White et al., 2017; Kondo et al., 2018). Affective benefits have been found for participants who experience different forms of nature exposure, including viewing nature images (Ulrich et al., 1991), or being physically present within the environment (Hartig et al., 2003; Berman et al., 2008). Study designs include cross-sectional and longitudinal cohort approaches (White et al., 2013; Mitchell et al., 2015; Wheeler et al., 2015; van den Berg et al., 2016), natural experiments (South et al., 2018), and controlled field and laboratory experiments (Ulrich et al., 1991; Hartig et al., 2003; Berman et al., 2012; Aspinall et al., 2015). Outcomes include both increased positive affect (Berman et al., 2008; Park et al., 2010) and decreased negative affect (Hartig et al., 2003; Bratman et al., 2015a).

It is not yet clear, however, what psychological mechanisms underlie these effects. One possibility is informed by prior work on the mood regulating potential of natural landscapes (Korpela et al., 2001; Gonzalez et al., 2010; Johnsen and Rydstedt, 2013). This work suggests that the affective benefits of nature contact might be due to shifts in emotion regulation that occur from nature exposure, including increased engagement in adaptive strategies, or decreased engagement in maladaptive ones. One example of a maladaptive form of emotion regulation that might be reduced by nature exposure is rumination (repetitive and negative self-referential thought about causes and consequences of mood that involves a detrimental pattern of attention allocation) (Nolen-Hoeksema et al., 2008; Roelofs et al., 2009; Aldao and Nolen-Hoeksema, 2010; Joormann and Gotlib, 2010; Genet and Siemer, 2012). Heightened rumination has been shown to be associated with increased negative affective outcomes that stem from real-life events (Moberly and Watkins, 2008; Genet and Siemer, 2012), and a greater risk of experiencing depressive episodes (Nolen-Hoeksema, 2000).

There are a number of possible pathways by which nature contact might reduce rumination. With respect to the framework of Attention Restoration Theory (ART; Kaplan, 1995), it is possible that rumination is affected by the restoration of directed attention, reduction of attentional fatigue, and/or improvement in cognitive control that comes from nature experience (Kaplan, 1995; Berto, 2005; Wells et al., 2019). Also in line with ART, nature contact could encourage the engagement of “soft fascination”—a cognitive process that may provide the mental bandwidth for reflection (Schertz et al., 2018; Basu et al., 2019). This engagement may then result in a decreased salience of the types of unresolved thought patterns that can be characteristic of rumination. Other pathways outside the framework of ART include the possibility that nature contact may provide a “positive distraction” away from a dwelling on negative aspects of well-being (Jiang et al., 2019). Or a change in contextual cues (such as billboards, advertisements, etc.) in natural vs. urban environments may provide a respite from reminders regarding the achievement of a goal, social comparisons, or habitual thought patterns that can be triggers for ruminative thought (Aldao, 2013; Watkins and Nolen-Hoeksema, 2014).

It is important to make a distinction between two areas of research with respect to nature contact and rumination. The first area involves an investigation into the ways in which the environment may impact the strength of association between rumination and negative affective outcomes that stem from real-life events (i.e., mood reactivity), or individual characteristics (such as state-level intensity and experience of chronic pain) (Wells et al., 2019). The second area involves an investigation into the association or impact of nature contact on individuals' levels of state or trait-level rumination (Gonzalez et al., 2010; Bratman et al., 2015a) (i.e., the ways in which nature exposure impacts or is associated with differences or changes in rumination). These streams of research are separate, though not mutually exclusive.

Here, we are focused on this second area of research—specifically, the ways in which average amounts of weekly nature contact may be associated with different levels of general tendencies to ruminate for individuals. The possibility that contact with the natural environment is associated with rumination is consistent with prior work in which participants randomized to a brief nature exposure reported decreases in rumination vs. participants randomized to a brief urban exposure (Bratman et al., 2015a,b). However, these findings occurred in studies with a short temporal scale (50- and 90-min, single-session walks) and sample sizes were limited. It is also not clear whether these findings generalize to naturally occurring variation in nature exposure over longer periods of time, and general tendencies to ruminate.

To be clear, our hypotheses assume that average weekly nature contact has the potential to influence a general tendency to engage in ruminative thought, which in turn can have affective consequences. Though some may consider this to be an assessment of dispositional rumination, and therefore an aspect of cognition and affective processing that is not likely to be associated with aspects of the environment with which one regularly interacts, we believe this relationship to be tenable and motivated by prior evidence and theory. In general, some environmental characteristics may encourage or trigger habitual ruminative thought (Watkins and Nolen-Hoeksema, 2014), while others may lack these elements, or instead provide characteristics that provide for restorative experiences (Korpela et al., 2008) or encourage a shift of attentional focus away from the self (Pasanen et al., 2018). It is quite conceivable that a regular engagement with specific environments would lead to a duration of these effects or attention allocation that could subsequently translate into a difference in self-reported levels of rumination in general.

Additionally, literature from environmental epidemiology and psychology supports this possibility for related outcomes, as exposure to natural environments on a regular basis has been shown to be associated with general levels of life satisfaction (Chang et al., 2020), depressive symptoms and depression (Shanahan et al., 2016; Fong et al., 2018), and even development of personality, namely the facets of Openness and Neuroticism (Snell et al., 2020). Here, we aimed to examine whether this association extended to general levels of rumination. To examine this issue, we conducted a cross-sectional study that examined associations among average time spent in nature, and general levels of rumination, as well as positive and negative affect.

For this study, we conducted a cross-sectional mediation analysis to test the hypotheses that:

(H1) Average weekly time spent in nature would be positively associated with levels of positive affect, and inversely associated with negative affect,
(H2) Average weekly time spent in nature would be associated with lower levels of rumination,
(H3) Average weekly time spent in nature would be significantly and indirectly related to positive and negative affect through lower levels of rumination, a relationship that is consistent with rumination mediating the relationship between nature exposure and affect.

## Methods

The study was approved by the Stanford University Human Subjects Committee. Participants were given course credit to participate in the study and signed informed consent. Instructions for student participation were posted online through a school recruitment website and to an electronic mailing list sent to enrolled students. Students completed questionnaires online, all of which were presented in random order across participants. The survey took place over the course of 16 months, thereby accounting for weather and seasonal variability. Additional measures not relevant to the present report were obtained, and will be presented elsewhere.

### Participants

A total of 617 enrolled students, age 18 and over, from the San Francisco Bay Area completed the study (see Table 1 for descriptive statistics). No other exclusionary criteria were included. Twelve participants were excluded from analysis due to a reporting of hours per week spent in nature that was greater than three standard deviations above the sample mean, leaving a total of 605 participants. As we were not sure of expected effect sizes, we collected data from as many participants as were available during the course of a 16-month period (*post-hoc* power analyses included in Table 1 in Supplementary Material).

**Table 1:** Summary statistics of study population (*N* = 605).

**Variable**	**Mean (SE) or percentage**	**SD**
Age	25.30 (0.34)	8.40
**Gender**		
Women	69.92%	
Men	29.92%	
Non-binary	0.17%	
**Ethnicity**		
African-American	6.28%	
Asian, Pacific-Islander	37.69%	
Hispanic	20.83%	
Native-American	0.83%	
White	33.55%	
Other	10.25%	
Hours in nature per week	8.17 (0.32)	7.93
RRS brooding subscale	2.36 (0.03)	0.71
PANAS positive affect subscale	3.42 (0.03)	0.77
PANAS negative affect subscale	2.15 (0.03)	0.79

*The ethnicity measure offered multi-option selection leading to a total percentage sum greater than 100*.

### Measures

#### Nature Experience

Amount of time spent in nature was operationalized using an open-ended question (“On average, approximately how many hours per week would you consider yourself to have interacted with nature? For example, walking outside, biking, gardening, playing games/sports, camping, fishing, reading outside, yard work, hanging out in a park, etc...”). Answers ranged from 0–100 h per week. After elimination of the 12 statistical outliers (defined *a priori* as 3 SD), answers ranged from 0 to 40 h per week.

#### Rumination

Rumination was assessed using a five-item rumination scale adapted from the Ruminative Responses Scale (RRS; Treynor et al., 2003). This scale consisted of five items designed to assess the brooding component of rumination (e.g., “I think ‘Why do I have problems other people don't have?”'). Each item was rated on a 4-point scale, ranging from one (“never”) to four (“always”). The scale was internally consistent (α = 0.83).

#### Positive and Negative Affect

The 20-item Positive and Negative Affect Schedule (PANAS; Watson et al., 1988) was used to assess both positive affect (10-item scale; e.g., “interested,” “active”) and negative affect (10-item scale; e.g., “distressed,” “irritable”). Each item consisted of 5-point ratings, ranging from one (“very slightly or not at all”) to five (“extremely”), for which participants were asked to self-report the extent to which they “generally experience each of these feelings or emotions.” Each of the two scales was internally consistent (positive affect α = 0.90; negative affect α = 0.90).

### Statistical Analyses

We used the lavaan package (version 0.6-6) in R (version 4.0.0) to conduct confirmatory factor analyses and structural equation modeling (Rosseel, 2012).

### Confirmatory Factor Analysis

For the confirmatory factor analysis (CFA), we examined the latent structures of rumination and affect. The rumination factor was composed of the original five scale items. Before fitting the CFA model, affect items were averaged to create three parcels (or indicators) per factor. The creation of parcels to serve as indicators is recommended when five or more items represent a single factor, as parcels are more normally distributed and reliable than individual items, and the use of fewer parcels (compared to the number of original items) results in a more parsimonious model that is consistent with classical test theory (see Little et al., 2002). Since different item-parcel allocations can result in variations in model fit statistics and parameter estimates, all subsequently reported model results are averaged over 50 random item-parcel allocations (Sterba, 2011).

### Structural Mediation Model

We tested the hypothesized mediation model between time in nature and affect through rumination using a structural equation modeling framework. Using structural equation modeling to test mediation is superior to the classic multiple regression approach due to both its practical flexibility, (e.g., allowing for the use of latent factors and simultaneous estimation of multiple paths) and its more accurate estimation of parameters as demonstrated in simulation studies (Iacobucci et al., 2007; Gunzler et al., 2013). This approach allowed us to examine each of our three hypotheses noted above. We evaluated model fit [i.e., root mean square error of approximation (RMSEA), comparative fit index (CFI), Tucker-Lewis index (TLI), and the standardized root mean square residual (SRMR)] according to existing guidelines (Hoyle and Panter, 1995; Hu and Bentler, 1999; Marsh et al., 2004). For RMSEA, values ≤ 0.05 and 0.08 indicate close and reasonable fit respectively. For CFI and TLI, values ≥ 0.95 and 0.90 indicate close and reasonable fit respectively. For SRMR, values ≤ 0.08 are considered good fit. We report bias-corrected, bootstrapped confidence intervals. To test the significance of the indirect effects (the ab path) of the mediation model, we used bootstrapped 95% confidence intervals (Mackinnon et al., 2004). To test the significance of all other paths, we used bootstrapped standard errors to derive a *p*-value (with a cutoff *p* < 0.05) under a standard normal distribution.

## Results

Our results supported our hypotheses regarding (1) the association of time in nature with affect, (2) the association of time in nature with rumination, and (3) the mediation of the association of time in nature with negative affect through rumination, while the association of time in nature with positive affect through rumination was marginally significant.

### Confirmatory Factor Analysis

The three-factor CFA model of rumination, positive affect, and negative affect had good fit as indicated by a significant chi-square and conventional fit statistics, χ^2^ = 180, *df* = 41, *p* < 0.0001, RMSEA = 0.07 [90% confidence interval (CI) = 0.06, 0.09], CFI = 0.96, TLI = 0.95, SRMR = 0.04. Standardized factor loadings were uniformly large (from 0.64 to 0.88) and statistically significant (*p* < 0.001). These results demonstrated that we were measuring our constructs appropriately, and could proceed with the structural equation modeling to examine total, direct, and indirect effects.

### Structural Equation Modeling

**Figure 1:**
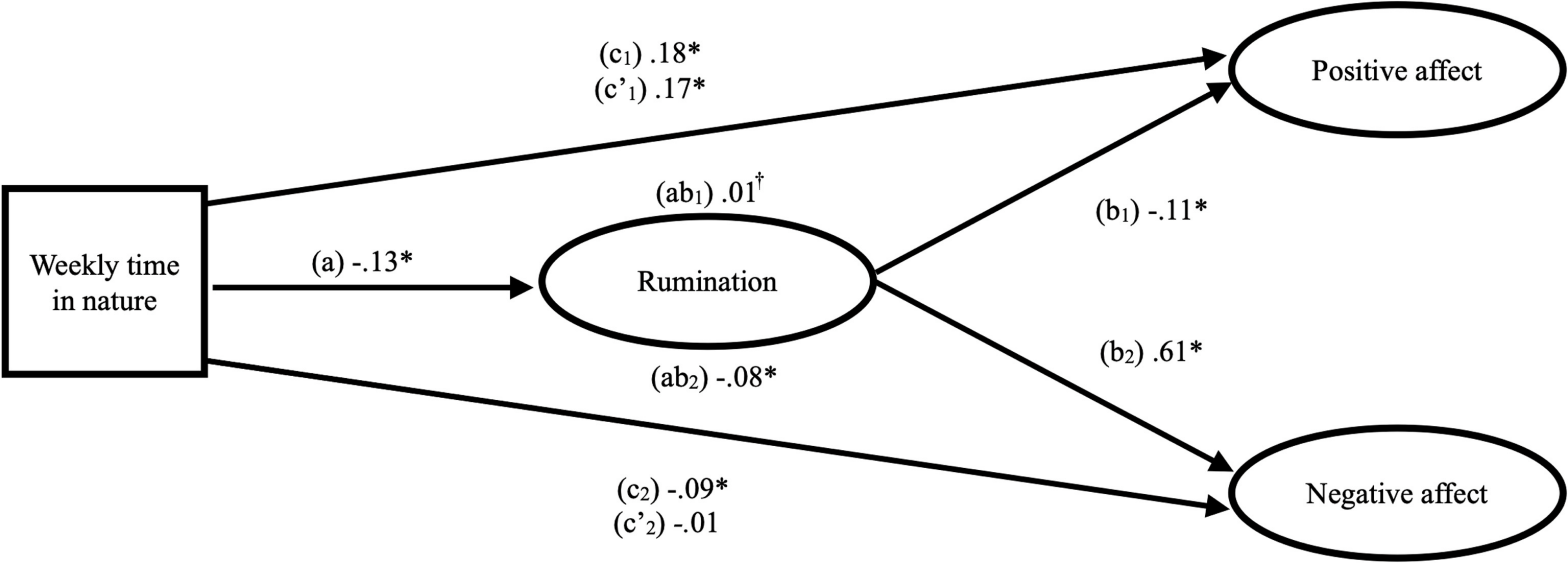
Structural equation mediation model of the relationships between time in nature, rumination and positive and negative affect. Ovals denote latent variables (i.e., all but “Weekly time in nature”). Standardized coefficients are presented. *indicates path with significant relationship (α = 0.05). †Indicates path with marginally significant relationship (α = 0.10). For the “ab” paths, significance was tested with bootstrapped 95% confidence intervals. The “ab” paths indicate the indirect effect of time in nature on affect. The “c” paths indicate the total effect of time in nature on affect. The “c”' paths indicate the direct effect of time in nature on affect.

As shown in Table 2, in support of H1, there was a significant positive total effect of time in nature on positive affect (c_1_ path) and a significant negative total effect of time in nature on negative affect (c_2_ path). In support of H2, time in nature was inversely associated with rumination (a path). In support of H3, rumination was inversely associated with positive affect (b_1_ path) and positively associated with negative affect (b_2_ path), and when accounting for rumination, the direct effect of time in nature on positive affect was virtually unchanged and still significant (c'_1_ path). Moreover, when accounting for rumination, the direct effect of time in nature on negative affect turned non-significant (c'_2_ path). Additionally, the 95% confidence interval for the indirect effect of time in nature on positive affect was marginally significant with a very small effect size (ab_1_ path; std β = 0.01; see Table 2 and Figure 1). The 95% confidence interval for the indirect effect of time in nature on negative affect through rumination was significant (ab_2_ path; std. β = −0.08; see Table 2 and Figure 1) and had a larger effect size than time in nature on positive affect. Bivariate correlations are reported in Table 2 in Supplemental Material.

**Table 2:** Estimates (Est.) and effect sizes (Std. β) for all paths in the mediation model.

		**Positive affect**			**Negative affect**		
**Effect**	**Path**	**Est. (SE)**	**Std. β**	***p*-value or 95% CI**	**Est. (SE)**	**Std. β**	***p*-value or 95% CI**
	a	−0.010 (0.003)	−0.13	0.001	0.010 (0.003)	−0.13	0.001
	b	−0.140 (0.069)	−0.11	0.046	0.813 (0.084)	0.61	<0.001
Direct	c'	0.016 (0.003)	0.17	<0.001	−0.001 (0.003)	−0.01	0.801
Total	c	0.017 (0.003)	0.18	<0.001	−0.009 (0.004)	−0.09	0.029
Indirect	a*b	0.001 (0.001)	0.01	[0.000, 0.003]	−0.008 (0.002)	−0.08	[−0.013, −0.003]

*SE = standard error; CI = confidence interval. Effect sizes are reported as Std. β. 95% confidence intervals were obtained by bootstrapping to test significance of indirect effects in accordance with Mackinnon et al. (2004)*.

### Secondary Analyses

We also ran a version of our model with demographic covariates. Before fitting this model, we removed the single non-binary individual and created binary variables for men/women and white/non-white ethnicity. We then followed the same model fitting procedure as in the main analysis with the addition of control variables for gender, ethnicity, and age on both the “a” path and the “b” paths of the mediation model. Our hypothesized model had good fit, χ^2^ = 252, *df* = 76, *p* < 0.0001, RMSEA = 0.06 [90% confidence interval (CI) = 0.05, 0.07], CFI = 0.95, TLI = 0.94, SRMR = 0.04. On the negative pathway, all predicted relationships and indirect effects observed in the previous model remained significant. On the positive pathway, the effect of rumination on positive affect (b_1_ path; std. β = −0.10, *p* = 0.090) turned non-significant. This appears to be due to a lack of power rather than a confounding effect since the effect size remained virtually unchanged. The indirect effect of time with nature on positive affect through rumination remained marginally significant and the effect size remained unchanged.

## Discussion

### Summary of Findings

Urbanization is taking place around the globe at an unprecedented rate. Over half of humanity now lives in urban areas, and projections suggest that this trend will continue over the next several decades (Dye, 2008). With this trend comes a marked decrease in the rate of regular contact with natural environments (Skår and Krogh, 2009), and there is growing evidence that there may be negative repercussions for human affective functioning due to the lack of nature experience that accompanies urbanization (for reviews see Bratman et al., 2012; Frumkin et al., 2017). However, little is known about the mechanisms underlying the affective impacts of nature experience.

In this study, we used structural equation modeling of cross-sectional data to test a hypothesized pathway of the affective benefits of nature experience through rumination. Our results supported our hypotheses that (H1) average weekly time spent in nature would be positively associated with levels of positive affect, and inversely associated with negative affect, (H2) average weekly time spent in nature would be associated with lower levels of rumination, and partially supported (H3) average weekly time spent in nature would be significantly and indirectly related to positive and negative affect through lower levels of rumination, insofar as we found a relationship that is consistent with rumination marginally significantly mediating the relationship between time in nature and positive affect, and significantly mediating the relationship between time in nature and negative affect. When accounting for rumination, the direct effect of time in nature on positive affect was virtually unchanged and still significant, whereas it was non-significant for negative affect. This marginal mediation for positive affect could be due to other factors that relate time in nature with positive affect, including but not limited to stress reduction through physiological pathways that bypass executive function (Ulrich et al., 1991). Although the magnitude of the total effect of time in nature on affect was larger along the positive affect pathway, it appears that the effect of time in nature on rumination is more consequential for negative affect. This may be due to the possibility that rumination is associated to a greater degree with negative vs. positive affect, or that different emotion regulation pathways underlie the relationship of nature contact with the distinct and often independent dimensions of negative and positive affect (Watson et al., 1988).

Although this study is cross-sectional, our findings suggest that nature experience may result in affective benefits *via* decreases in rumination, especially through the mediation of negative affect. Why might nature exposure lead to changes in rumination? Three mutually compatible possibilities merit consideration.

First, in line with ART, nature experience may result in a replenishment of directed attention, an engagement of “soft fascination,” increased cognitive control, and an increased ability to reflect (Berto, 2005; Schertz et al., 2018; Basu et al., 2019; Wells et al., 2019). A subsequent broadening of attentional scope may follow, thereby potentially decreasing rumination through the facilitation of greater access to more semantic information (Grol et al., 2015). Additionally, through pleasantness, novelty, or other aesthetic characteristics, it is possible that natural environments may encourage a distraction away from the self—a strategy that has been shown to decrease rumination and anxiety (Nolen-Hoeksema et al., 2008). Prior work has hypothesized that nature may provide opportunity for this “positive distraction” (Jiang et al., 2019).

Second, according to control theory, repetitive thought may be initiated with the intention of promoting progress toward the *achievement of a goal* (Martin and Tesser, 1996). This repetitive thought can become ruminative in cases in which an individual is focused on the discrepancy between a current state and the desired state of having achieved the goal, while not making progress toward its achievement (Watkins, 2008). The focus of attention on this discrepancy can perpetuate a sense of incompleteness and lack of resolution, leading to increased negative affect. This negative affect can, in turn, increase a belief that progress has not been made toward goal achievement (Chan et al., 2013). This pattern of rumination may continue, unless and until a disengagement with the underlying goal is attained (Watkins, 2008), as the individual may falsely believe that the rumination is constructive and helpful in achieving the goal, when this is not in fact the case (Hawksley and Davey, 2010). With regard to nature experience, natural landscapes may differ from urban ones with respect to environmental cues (e.g., advertisements, cues regarding social hierarchy) or reminders of these goals (i.e., comparatively fewer of these cues may exist in natural environments). Nature may thus encourage a disengagement from goal pursuit, thereby decreasing ruminative repetitive thought. Additionally, our finding of an association of nature experience with positive affect may have implications in this sense, as positive affect may increase the likelihood that an individual feels he or she has achieved a goal (Hawksley and Davey, 2010; Chan et al., 2013).

Third, a *change in context* from an urban environment to a natural one may also have repercussions for conditioned, habitual thinking patterns that are consciously or unconsciously reinforced by environmental stimuli (Aldao, 2013). The habit-goal framework (Watkins and Nolen-Hoeksema, 2014) of depressive rumination posits that the types of ruminative, repetitive thoughts associated with goal discrepancies that are outlined above may become habitual – and that an “automatic association” can be formed between this particular response and the context (e.g., a physical location) in which it occurs (Watkins and Nolen-Hoeksema, 2014). In line with operant conditioning and associative learning, a response-outcome association can be contingent upon a given context. Eventually the behavioral response may be triggered automatically by the context, regardless of the salience of the goal. With this model in mind, it seems possible that for an individual for whom a habit of ruminative thought is formed in an urban or suburban context, a change in context to a natural environment could provide an atmosphere that breaks such habitual patterns of thought.

## Conclusions and Limitations

Our findings suggest that average weekly nature contact is associated with general affect and rumination, and that the associations of nature contact with negative affect (and potentially positive affect) may be explained at least in part through a decreased tendency to engage in maladaptive emotion regulation (i.e., rumination). This represents a step forward in the exploration of the relationship between environment and emotion-regulatory strategies. Given our uncertainty regarding effect sizes, we opted for the use of a cross-sectional, large-N survey. Although our findings of mediation are consistent with a causal account in which nature exposure has its effects on affective outcomes *via* rumination, the cross-sectional nature of the current data preclude causal inferences, and additionally the effect size of the indirect effect for positive affect was very small. However, given our measures of average contact with nature and general levels of rumination, it is also possible that the strength of the association is in fact an underestimation, and that the short-term impact on rumination from nature contact is more pronounced than the magnitude of our coefficients indicate. In future work, it will be important to extend the present findings in longitudinal, experimental studies that allow for the establishment of a temporal sequencing and causal pathways among variables. Additionally, although we attempted to collect as many responses as possible over a 16-month period, future studies should endeavor to conduct formal power analyses based on available evidence. It is also interesting to note that the size of the direct effect of nature contact on positive affect was small/moderate, and that this effect remained virtually unchanged after accounting for the small indirect effect *via* rumination. Future work should examine whether the remaining unexplained direct effect of time in nature on positive affect travels *via* other emotion regulation strategies or by other mechanisms entirely.

A second important direction for future research is to expand the types of participants included. Our sample was restricted to college students from the San Francisco Bay Area, thus limiting the generalizability of our findings. In future work, it will be important to consider the impact of nature experience on affect for individuals from community samples, including participants with clinically significant variation in affect, such as those with anxiety or depressive disorders.

A third research direction is to further clarify the pathway by which emotion regulation mediates the link between nature exposure and affective outcomes. For example, it is possible that general tendencies to ruminate mediate nature visitation through experiential avoidance and behavioral withdrawal—individuals with high levels of dispositional rumination may experience difficulty in visiting or engaging in nature contact, consistent with its role in depression (Cribb et al., 2006; Dickson et al., 2012). This in turn could impact the degree to which individuals feel able to visit and benefit from the restorative and self-regulating aspects of nature (Korpela et al., 2018, 2020)—a form of situation selection that could have negative affective consequences. Future research should also consider a broader set of emotion regulation processes in order to more clearly delineate which emotion regulation strategies play crucial roles and other, adaptive modes of emotion regulation as well. For example, Panno et al. (2020) found that higher levels of self-reported frequency of use of cognitive reappraisal was associated with individuals' experience of “being away” in natural environments.

A fourth direction for future research is suggested by our measure of nature exposure. This measure was designed to limit participant burden, and it therefore lacks information on frequency and type of nature exposure. Our concerns on this front are tempered by the fact that other studies support the validity of using a single-item measure, in particular with respect to the focus on duration, as it relates to overall subjective well-being. White et al. (2019) found an association of nature exposure duration (calculated as weekly nature contact reported in 60-min blocks) with self-reported health and well-being. And Shanahan et al. (2016) found that prevalence of depression was associated with the aspect of their nature contact assessments that was tied to duration (also measured in weekly minutes per week). Additionally, in separate analyses we found that our single-item measure correlates strongly with these items from Shanahan et al. (2016) (see Supplemental Material)—further indicating that our specific measure is capturing duration of nature contact, as well as some of the predictive power of visit frequency. Future work should further investigate the specific affective and emotion regulation outcomes that are associated with duration vs. frequency of nature contact, as well as the forms of interaction (e.g., active vs. passive exposure) and natural elements with which this contact occurs (Kahn et al., 2010; Holt et al., 2019). The wording of our measure could also be further examined and potentially adjusted in future work to increase certainty that it specifically relates to exclusively natural environments, and to allow for a comparison of more vs. less physically active types of nature contact, as some work has shown that this may mediate the association of nature exposure with well-being, though this evidence is mixed (de Vries et al., 2013; Dadvand et al., 2016; Frumkin et al., 2017). Finally, it will be important to examine the ways in which different types of nature impact different people differently, and how different aspects of the urban environment moderate the impacts of these effects on well-being (Keniger et al., 2013; Sullivan et al., 2014; Bratman et al., 2019; Keeler et al., 2019). Future work could also integrate lengthier measures of rumination than our five-item assessment, though it is a validated measure and showed good internal consistency.

In the present research, we focused on rumination as a mediator of the impact of nature experience on affective responding. We believe that the present findings support and inform an important, emerging area of research at the nexus of environment and emotion regulation. Future work should expand upon these results and continue to investigate the ways in which nature may be incorporated into the broader frameworks that consider the social and environmental predictors of human health that are relevant to urban planning.

## Data Availability Statement

The raw data supporting the conclusions of this article will be made available by the authors, without undue reservation.

## Ethics Statement

The studies involving human participants were reviewed and approved by Stanford University Human Subjects Committee. The patients/participants provided their written informed consent to participate in this study.

## Author Contributions

GB, GY, IL, GD, and JG conceived of and conducted the study. AM and IL completed data analyses. GB and GY collected all data. GB, GY, AM, IL, GD, and JG contributed to framing and writing the manuscript. All authors contributed to the article and approved the submitted version.

## Conflict of Interest

The authors declare that the research was conducted in the absence of any commercial or financial relationships that could be construed as a potential conflict of interest.
