# Supplementary material for: Affective Benefits of Nature Contact: The Role of Rumination
Source: Front Psychol. 2021 Mar 10;12:643866. doi: 10.3389/fpsyg.2021.643866 (PMC7988226; doi:10.3389/fpsyg.2021.643866)
Supplement: Supplementary file 1 [file Table_1.DOCX]

**Post-hoc power analysis.** To conduct a post-hoc power analysis, we used the R package *simsem* (version 0.5.15; Pornprasertmanit et al., 2020). For each of the 50 fitted models (each constructed with random parcel assignments), we simulated 1000 datasets that conform to the parameters of the model. We then calculated the power (i.e., percentage of times the null hypothesis was rejected at *p* < .05) of each model parameter. In Supplemental Table 1, we report the power for each model parameter averaged over each of the 50 model variations.

**Supplemental Table 1**

Power for each path in the mediation model

| Path | Variables | Power |
| --- | --- | --- |
| a | Nature – Rumination | 84.16% |
| b_1_ | Rumination – Positive affect | 65.07% |
| b_2_ | Rumination – Negative affect | >99.99% |
| ab_1_ | Nature – Rumination – Positive affect | 31.96% |
| ab_2_ | Nature – Rumination – Negative affect | 83.90% |
| c’_1_ | Nature – Positive affect | 96.99% |
| c’_2_ | Nature – Negative affect | 6.92% |
| c_1_ | Nature – Positive affect | 98.61% |
| c_2_ | Nature – Negative affect | 54.84% |

**Supplemental Table 2**

Means, standard deviations, and correlations with confidence intervals

| Variable | *M* | *SD* | (1) | (2) | (3) |
| --- | --- | --- | --- | --- | --- |
|  |  |  |  |  |  |
| 1. Negative affect | 2.15 | 0.79 |  |  |  |
|  |  |  |  |  |  |
| 2. Positive affect | 3.42 | 0.77 | -.08* |  |  |
|  |  |  | [-.16, -.00] |  |  |
|  |  |  |  |  |  |
| 3. Rumination | 2.36 | 0.71 | .53** | -.10* |  |
|  |  |  | [.47, .58] | [-.18, -.02] |  |
|  |  |  |  |  |  |
| 4. Nature hours/week | 8.17 | 7.93 | -.08* | .17** | -.12** |
|  |  |  | [-.16, -.00] | [.10, .25] | [-.20, -.04] |
|  |  |  |  |  |  |

*Note.* *M* and *SD* represent mean and standard deviation, respectively. Values in square brackets indicate the 95% confidence interval for each correlation. * indicates *p* < .05. ** indicates *p* < .01. Negative affect indicates the mean score on the negative subscale of the PANAS. Positive affect indicates the mean score on the positive subscale of the PANAS. Rumination indicates the mean score on the RRS. Nature hours/week indicates the average number of hours per week spent in nature.

**Nature contact measure correlation.** We conducted a survey of 98 North American participants (after removal of failed attention checks (N=26) and outliers (N=2; a priori defined as +3SD self-reported number of hours of nature contact per week), in which we calculated the Spearman’s rho correlation between our measure and an ordinal measure of frequency of nature contact over the past year, as well as duration of nature contact over the past week, both from (Shanahan et al., 2016). There was a strong positive correlation between our item and their measure’s assessment of *frequency (over past year)* measure (r_s_ = .41, p < .0001). There was also a strong positive correlation between our item and their measure’s assessment of *duration (over past week)* measure (r_s_ = .50, p < .0001).

**References**

Pornprasertmanit, S., Miller, P., Schoemann, A., Quick, C., Jorgensen, T., and Pornprasertmanit, M. S.

(2020). Package ‘simsem’. Acquired from

https://cran.r-project.org/web/packages/simsem/simsem.pdf

Shanahan, D. F., Bush, R., Gaston, K. J., Lin, B. B., Dean, J., Barber, E., and Fuller, R. A. (2016). Health benefits from nature experiences depend on dose. *Scientific Reports*, *6*(1), 28551-28551. <https://doi.org/10.1038/srep28551>
